# Membrane Vesicles Released by a hypervesiculating *Escherichia coli* Nissle 1917 *tolR* Mutant Are Highly Heterogeneous and Show Reduced Capacity for Epithelial Cell Interaction and Entry

**DOI:** 10.1371/journal.pone.0169186

**Published:** 2016-12-30

**Authors:** Carla Pérez-Cruz, María-Alexandra Cañas, Rosa Giménez, Josefa Badia, Elena Mercade, Laura Baldomà, Laura Aguilera

**Affiliations:** 1 Secció de Microbiologia, Departament de Biologia, Sanitat i Medi Ambient, Facultat de Farmàcia, Universitat de Barcelona, Barcelona, Spain; 2 Secció de Bioquímica i Biologia Molecular, Departament de Bioquímica i Fisiologia, Facultat de Farmàcia, Universitat de Barcelona, Barcelona, Spain; 3 Institut de Biomedicina de la Universitat de Barcelona, Barcelona, Spain; Hudson Institute, AUSTRALIA

## Abstract

Membrane vesicles (MVs) produced by Gram-negative bacteria are being explored for novel clinical applications due to their ability to deliver active molecules to distant host cells, where they can exert immunomodulatory properties. MVs released by the probiotic *Escherichia coli* Nissle 1917 (EcN) are good candidates for testing such applications. However, a drawback for such studies is the low level of MV isolation from *in vitro* culture supernatants, which may be overcome by the use of mutants in cell envelope proteins that yield a hypervesiculation phenotype. Here, we confirm that a *tolR* mutation in EcN increases MV production, as determined by protein, LPS and fluorescent lipid measurements. Transmission electron microscopy (TEM) of negatively stained MVs did not reveal significant differences with wild type EcN MVs. Conversely, TEM observation after high-pressure freezing followed by freeze substitution of bacterial samples, together with cryo-TEM observation of plunge-frozen hydrated isolated MVs showed considerable structural heterogeneity in the EcN *tolR* samples. In addition to common one-bilayer vesicles (OMVs) and the recently described double-bilayer vesicles (O-IMVs), other types of MVs were observed. Time-course experiments of MV uptake in Caco-2 cells using rhodamine- and DiO-labelled MVs evidenced that EcN *tolR* MVs displayed reduced internalization levels compared to the wild-type MVs. The low number of intracellular MVs was due to a lower cell binding capacity of the *tolR*-derived MVs, rather than a different entry pathway or mechanism. These findings indicate that heterogeneity of MVs from *tolR* mutants may have a major impact on vesicle functionality, and point to the need for conducting a detailed structural analysis when MVs from hypervesiculating mutants are to be used for biotechnological applications.

## Introduction

Commensal and pathogenic Gram-negative bacteria have evolved different systems to contact host cells. One mechanism is the formation of membrane vesicles that can deliver the cargo to distant targets in the host [1]. Bacterial membrane vesicles (MVs) are spherical membranous structures with diameters ranging between 20 and 300 nm. Produced during the normal growth of Gram-negative bacteria, they enable a protected secretion of proteins, lipids, RNA, DNA and other effector molecules [2,3]. Many studies with Gram-negative pathogens conducted in the last decade have shown that MVs are internalized in host cells and contribute to virulence by delivering cytotoxic factors as well as mediators that interfere with the immune system [4,5]. When first discovered, MVs from pathogenic bacteria were proposed as vaccines, and research in this field continues [6–8]. Promising novel therapy applications include using engineered MVs expressing antigens from pathogenic strains or as specialized drug delivery vehicles [9,10].

One drawback for functional and applied studies with MVs is the low yield of vesicles recovered from *in vitro* culture supernatants. Different strategies have been assayed to improve yields, such as growing bacteria under stressed conditions, in the presence of antibiotics, or the use of mutants in components of the cell envelope [11–15]. MV formation takes place after the outer membrane is detached from the peptidoglycan (PG) located in the periplasmic space. For this reason, crosslinking of the PG with membrane components is needed for cell stability and has been studied extensively. The PG interacts with the outer membrane porin OmpA and with the Tol-Pal protein complex, and establishes covalent cross-linking with Brauns’s lipoprotein (Lpp). Under natural conditions, changes in the interaction between these envelope components without disturbance of the membrane stability are described as crucial for MV biogenesis. With the aim of increasing MV production, different groups have obtained mutants in genes encoding cell envelope proteins. Thus, *ompA* mutants of *Escherichia coli*, *Vibrio cholerae*, and *Acinetobacter baumannii* [16–18], as well as *tol-pal* mutants of *E*. *coli* and *Helicobacter pylori* [19,20] have been reported as “hypervesiculating” strains, suitable for a high production of MVs under different growth conditions. A recent study analyzing MV production by the mutant strains of the Keio Collection identified around 150 genes involved in the vesiculation process. It was shown that mutations altering outer membrane structures generally lead to hypervesiculation phenotypes [21].

There is a need to characterize and quantify the MVs obtained from over-producing phenotypes. Different methods have been used to measure vesiculation levels but generally without clarifying the MV structure and composition [1]. In most published studies, MV morphology and integrity is revealed by transmission electron microscopy (TEM) micrographs from negatively stained MVs [13,19,22,23]. Although this technique is useful to confirm the presence of MVs, the resolution is insufficient to visualize irregular or atypical MVs, which may be obtained when working with genetically manipulated strains. Hypervesiculating mutants can produce atypical MVs, which may have surface antigens with a different conformation or display altered immunogenicity, self-adjuvation, or uptake by host cells. The variability caused by these features can affect studies evaluating the application of MVs in different fields [8–10].

In recent years, improvements in TEM and cryo-TEM techniques have enabled the imaging of biological specimens with greatly enhanced resolution. TEM observation of specimens cryoimmobilized by High Pressure Freezing (HPF) followed by Freeze Substitution (FS) and sectioning, together with cryo-TEM observation of frozen-hydrated specimens, allow visualization of biological samples close to their native state, enabling us to refine our knowledge of bacterial structures [24,25]. These techniques enabled us to visualize the formation of a new type of MVs in environmental and pathogenic bacteria [26,27], and may therefore be useful to characterize the fine structure of MVs from hypervesiculating strains.

*Escherichia coli* Nissle 1917 (EcN) is a probiotic used for the treatment of intestinal disorders. Its MVs modulate the cytokine /chemokine response of epithelial and immune cells in different *in vitro* and *ex vivo* models [28]. Moreover, engineered MVs derived from EcN are being analyzed as recombinant subunit antigen carriers for the development of pathogen-mimetic vaccines [29]. In this work, we constructed a *tolR* mutant derived from this probiotic strain. We analyzed the growth and vesiculation capacity of the EcN *tolR* mutant, and structurally characterized its MVs. For this purpose, wild type and *tolR* mutant EcN, as well as their derived MVs, were analyzed by TEM after HPF-FS and cryo-TEM. MV uptake by Caco-2 cells was analyzed as a functional parameter to evaluate whether EcN *tolR*-derived MVs are efficiently internalized by intestinal epithelial cells.

## Materials and Methods

### Bacterial strains and cell growth

The probiotic strain EcN (serotype O6:K5:H1) was provided by Ardeypharm GmbH (Herdecke, Germany). The mutant strain EcN *tolR* was constructed in this study by P1-transduction from *E*. *coli* strain TPS300 (*tolR*::Ω*cm*). Gene disruption was confirmed by PCR (S1 Fig). This mutation does not cause polarity to downstream *tolAB* genes [30]. For growth monitoring and MV production, EcN and EcN *tolR* were routinely grown at 37°C in Luria-Bertani broth (LB) in an orbital shaker at 250 rpm. Chloramphenicol (Cm) was added to the medium at 20 μg/ml final concentration. For HPF-FS methods, cells were grown on Trypticase Soy Agar (TSA, Oxoid) for 18 h at 37°C. Growth was monitored by measuring the optical density (OD) at 580 nm. Bacterial cells were counted by plating serial dilutions on LB agar plates.

### Isolation of MVs

MVs were isolated from culture supernatants as described previously [31]. Briefly, bacterial cells were grown aerobically in LB for 15 h and pelleted by centrifugation at 10,000 x *g* for 20 min at 4°C. The supernatants were filtered through a 0.22 μm-pore-size filter (Millipore) to remove residual bacteria and concentrated by centrifugation in a 100K Centricon^®^ Plus-70 filter device (Millipore), followed by an additional filtration step. MVs were recovered by centrifugation at 150,000 × *g* for 1 h at 4°C, washed and resuspended in phosphate buffered saline (PBS). MVs were again pelleted (150,000 × *g*, 1 h) and finally resuspended in an adequate volume of PBS. Sterility of samples was assessed on TSA agar plates.

### Quantification of MVs

MVs produced by EcN and EcN *tolR* from 1L of culture were quantified by three different methods. Protein concentration was measured by the Lowry method [32]. LPS content was quantified by the Purpald assay as described previously, using KDO as a standard [33]. Lipid content associated with MVs was determined using the lipophilic fluorescent dye FM4-64 (Thermofisher) as described previously [34]. Briefly, a portion of the sterile resuspended MVs was incubated with FM4-64 (5 μg/ml in PBS) for 10 min at room temperature. MVs alone and the FM4-64 probe alone were used as negative controls. After excitation at 515 nm, emission at 640 nm was measured with the multiplate reader SYNERGY HT (Biotek). Fluorescence was normalized by colony forming units (CFU), determined by the dilution plating method. All quantifications were done by triplicate in three independent experiments.

### Protein identification by LC-MS/MS

MV samples (10 μg protein) were separated by 10% sodium dodecyl sulfate polyacrylamide gel electrophoresis (SDS-PAGE) [35], and protein bands were visualized by staining with Sypro^®^ Ruby Protein Gel Stain (Molecular Probes^™^), following the protocol of the manufacturer. Differential protein bands between samples were excised for protein identification at the Proteomic Platform at The Scientific Park of Barcelona. Protein bands were digested with trypsin and analyzed in a NanoAcquity (Waters) coupled to LTQ-Orbitrap Velos (Thermo Scientific) mass spectrometer essentially as described previously [31]. Data were acquired in raw data format using the software Thermo Xcalibur (v.2.2). A database was created by merging the protein entries present in the public database Swiss-Prot *E*. *coli* (v. 10/10/2016) with a database containing all entries for *E*. *coli* Nissle 1917 from NCBI (v. 10/10/2016). The raw files obtained in the mass spectrometry analyses were used to search the database described above. The software used was Proteome Discoverer (v.1.4.1.14), with Sequest HT as the search engine. Both target and decoy databases were searched to obtain a false discovery rate (FDR) in order to discriminate between correct and incorrect peptide spectrum matches, using the same q-values as previously described [31]. The lists of identified proteins for each sample are provided as Supporting Information (S1 Table).

### Western Blotting of Lipopolysaccharide (LPS)

Western blot analysis of LPS was performed as described previously, using specific antibodies against *E*. *coli* LPS (Abcam) (1:5,000 dilution, overnight at 4°C) and donkey anti-rabbit immunoglobulin horseradish peroxidase-linked (GE Healthcare) as a secondary antibody (dilution 1:15,000, 1 h at room temperature) [28]. The protein-antibody complex was visualized using the ECL Plus Western blotting detection system (GE Healthcare).

### Negative staining and TEM

Isolated MVs were examined by TEM after negative staining as described previously [36]. Formvar/carbon-coated copper grids were activated by UV light. Isolated MVs were adsorbed on grids for 1 min and then washed with distilled water. Grids were stained with 2% uranyl acetate for 1 min. After rinsing, grids were viewed with a Tecnai Spirit electron microscope (FEI Company, Netherlands) at an acceleration voltage of 120 kV.

### TEM observation after HPF-FS

TEM observation of EcN and EcN *tolR* strains after HPF-FS was performed as described previously [34]. For this purpose, cells were grown on TSA agar plates at 37°C for 18 hours. Briefly, bacterial colonies were cryoimmobilized using a Leica EMPact high-pressure freezer (Leica, Austria), and were freeze-substituted in a Leica EM automatic freeze substitution (AFS) system (Leica, Austria), where the substitution was performed in pure acetone containing 2% (w/v) osmium tetroxide and 0.1% (w/v) uranyl acetate. Samples were embedded in Epon 812 (Ted Pella, Inc.). Epon-embedded thin sections were examined in a Tecnai Spirit electron microscope (FEI Company, Netherlands) at an acceleration voltage of 120 kV.

### Cryo-TEM analysis of isolated MVs

For cryo-TEM observation of isolated MVs, samples were prepared as described above for MV isolation from liquid cultures. Cryo-TEM analysis was performed as described previously [27]. Briefly, MV suspensions (5 μl) were applied on freshly glow-discharged Quantifoil R 2/2 grids (Quantifoil Micro Tools GmbH, Germany) and allowed to adhere for 4 min. The samples were vitrified using a Vitroblot (FEI Company, Netherlands), and were transferred to a Tecnai F20 microscope (FEI Company, Netherlands), using a Gatan cryotransfer system (Gatan Inc. CA, USA). Cryo-TEM visualizations were carried out at a temperature between −170°C and −175°C and at the accelerating voltage of 200 kV. Images were acquired using low-dose imaging conditions and an Eagle 4k x 4k Images charged-coupled device (CCD) camera (FEI Company, Netherlands).

#### MV internalization by Caco-2 cells

The human colonic cell line Caco-2 (ATCC HTB-37) was obtained from the American Type Culture Collection. Cells were cultured in Dulbecco’s Modified Eagle Medium (DMEM) High Glucose supplemented with 10% (v/v) fetal bovine serum, 25 mM HEPES, 1% non-essential amino acids and penicillin (100 U/ml) and streptomycin (100 μg/ml) (Gibco BRL). Cultures were incubated at 37°C with 5% CO_2_.

To monitor MV internalization in intestinal epithelial cells, MVs were fluorescently labelled with octadecyl rhodamine B-R18 (Life Technologies) as described previously [28]. MVs, purified as described above, were washed with PBS, resuspended in labelling buffer (50 mM Na_2_CO_3_, 100 mM NaCl, pH 9.2) in the presence of 1 mg/ml octadecyl rhodamine B-R18 and incubated for 1 h at 25°C. Labelled MVs were pelleted by centrifugation at 150,000 × *g* for 1 h at 4°C, resuspended in PBS (0.2 M NaCl) and washed twice to fully remove the unbound dye. After a final centrifugation step, the rhodamine-labelled MVs were resuspended in PBS (0.2 M NaCl) containing a protease inhibitor cocktail (Complete Protease Inhibitor Tablet, Roche) and stored at 4°C for up to 6 weeks.

MV internalization assays were performed using Caco-2 cells (18–20 days post-confluence) grown in a 96-well black plate (Corning Incorporated, Costar^®^). Prior to the assay, the medium was replaced with rhodamine B-R18-labelled MVs (1 μg protein/well) suspended in DMEM medium in the absence of phenol red and FCS. Cells were incubated at 37°C and fluorescence was measured over time using a Modulus^™^ Microplate Fluorometer (Turner BioSystems) (Ex 570 nm; Em 595 nm). Fluorescence intensity was normalized by the fluorescence of labelled MVs in the absence of epithelial cells. To determine the mechanism involved in the internalization process, Caco-2 cells were pre-treated with the endocytosis inhibitors chlorpromazine (15 μg/ml) or filipin III (10 μg/ml) for 1 h at 37°C prior to the addition of labeled-MVs. Control cells were not treated with the inhibitors.

MV internalization was assessed by confocal fluorescence microscopy as previously described [28,37]. Briefly, Caco-2 cells were grown in an 8-well chamber slider (ibidi) and incubated with rhodamine B-R18-labelled MVs (1 μg) at 37°C for 1 h, and then washed with PBS. Nuclei were labelled with DAPI. To visualize cell boundaries, the peripheral zonula occludens ZO-1 protein was stained using anti-ZO-1 rabbit IgG antibody (Invitrogen) and Alexa Fluor 488-conjugated goat anti-rabbit IgG (Invitrogen). Confocal microscopy was carried out using a Leica TCS SP5 laser scanning confocal spectral microscope with a 63x oil immersion objective lens. Images were captured with a Nikon color camera (16 bit). Fluorescence was recorded at 405 nm (blue; DAPI), 488 nm (green; Alexa Fluor 488), and 546 nm (red; rhodamine B-R18). Z-stack images were taken at 0.5 μm. Images were analyzed using the Fiji image processing package.

For flow cytometry analysis, MVs were labelled with 1% (v/v) fluorescence dye 3–3’-dioctadecyloxacarbocyanine perchlorate (DiO; Molecular Probes) as described elsewhere [38]. Caco-2 cells grown to confluence in 12-well tissue culture plates were trypsinized, resuspended in fresh DMEM without phenol red, and incubated at 37°C with Dio-labelled MVs (100 μg) for up to 4 h. To estimate the proportion of internalized MVs, extracellular vesicle fluorescence was quenched with trypan blue (0.25%). This treatment allows detection of only intracellular MVs [38]. At the indicated times, samples were taken and fluorescence intensities were measured using a Beckman Coulter Cytomics FC500 flow cytometer before (total amount of cell-associated MVs) and after (internalized MVs) the addition of trypan blue. Cell debris and dead cells were excluded from analysis by gating cells using FSC *vs* SSC double dot. A total of 10,000 events were analyzed for each gated sample. Mean fluorescence intensity values of untreated cells were subtracted from the values of MV-treated cells.

#### Cell viability assay

The trypan blue exclusion test was used to evaluate the effect of MVs on cell viability as described previously [37]. Caco-2 cells plated into 24-well plates were exposed to 5 μg/ml MVs for up to 168 h. As a rule, once every two days, the cells were trypsinized, stained with 0.25% w/v trypan blue, and counted with a haemocytometer.

## Results

### A *tolR* mutation in EcN increases MV production

The Tol-Pal system of *E*. *coli* is important for the maintenance of outer membrane integrity. It has been described that mutations in any of the *tol-pal* genes confer a defect in the outer membrane that leads to increased MV production [19–21].

**Fig 1:**
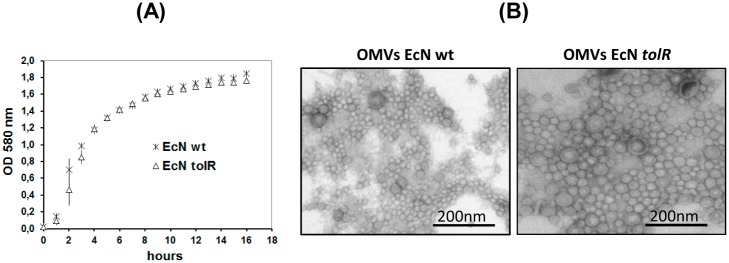
Effect of a *tolR* mutation on EcN growth and vesicle size. **(A)** Growth curves of wild-type EcN (cross) and EcN *tolR* (triangles) cultivated in LB medium. Values are means ± standard error from three independent experiments. **(B)** Negative staining electron microscopy of MVs released by these strains after 15 h growth in LB. For both strains, MVs were collected from a 1-litre culture and resuspended in a final volume of 0.2 ml. Representative images of MV samples from wild-type EcN (direct inspection) and EcN *tolR* (1:20 dilution) are shown. Scale bars: 200 nm.

To improve the MV yield of the probiotic EcN, we constructed a derived *tolR* defective mutant by P1 transduction. The donor *E*. *coli* strain was TPS300, which carries a Cm^r^ cassette insertion in the *tolR* gene (*tolR*::Ω*cm*) [30]. The mutation was confirmed by PCR (S1 Fig). To analyze the effect of the *tolR* mutation on cell growth and viability, growth curves of the wild-type and mutant strains in LB were measured, and their log-phase doubling times were calculated. Growth was monitored both by measuring the OD at 580 nm (Fig 1A) and counting viable cells on LB agar plates (S2 Fig). EcN *tolR* exhibited a slightly higher doubling time (23.3 min) than the wild-type EcN strain (19.3 min). However, no differences between strains were observed in the early stationary phase. The vesiculation levels of EcN and EcN *tolR* were compared after 15 h of growth, when both strains achieved an OD_580_ of 1.7 and cell counts of 4 x 10^9^ CFU/ml. MVs were isolated from cell-free culture supernatants and evaluated by negative stain-TEM. Images showed spherical MVs, which in the wild-type strain ranged in size from approximately 20 to 60 nanometers in diameter (Fig 1B). The *tolR* mutant MVs appeared to be larger (from 20 to 150 nm) and displayed greater variability in size, with a lower population of small MVs (Fig 1B).

MV production was estimated by three different methods: protein quantification, LPS concentration, and fluorescence measured after incubation with the lipid probe FM4-64. Protein and LPS were normalized for the volume of the culture from which MVs were isolated (1L), and fluorescence was normalized for the amount of bacteria (CFU). Results of MV quantification are shown in Table 1. All the three methods, revealed statistically significant differences in vesiculation between the mutant and the wild-type strains, with 32.9-, 51.95- and 77.78-fold increases for protein, LPS and fluorescence determinations, respectively. Although the protein content was higher in the *tolR* mutant, the lipidic content was far higher, and the protein to fluorescence ratio for wild-type and mutant strains gave values of 362.54 and 157.48, respectively.

**Table 1:** MV production of EcN and EcN *tolR* strains determined by three methods.

	EcN	EcN *tolR*	Δ MVs
Protein^a^	0.42 ± 0.03	13.01 ± 1.42	32.9* ± 7.04
LPS^b^	0.11 ± 0.04	5.20 ± 2.04	51.95* ± 29.72
RFU/CFU^c^	1.16 E-03 ± 3.7 E-04	8.26 E-02 ± 8.5 E-03	77.78* ± 22.03

Values are the means ± standard error from three independent experiments.

^a^ expressed as mg/L culture

^b^ expressed as mmol KDO/L culture

^c^ MVs lipid fluorescence was measured after incubation with FM4-64, and expressed as relative fluorescence units(RFU)/ by colony forming units (CFU)

Δ MVs, means increase in EcN *tolR* vesicle production relative to EcN.

* differences are statistically significant (p<0.05) according to Krustal-Wallis H test.

### Protein profiles of MVs produced by EcN and EcN *tolR*

**Fig 2:**
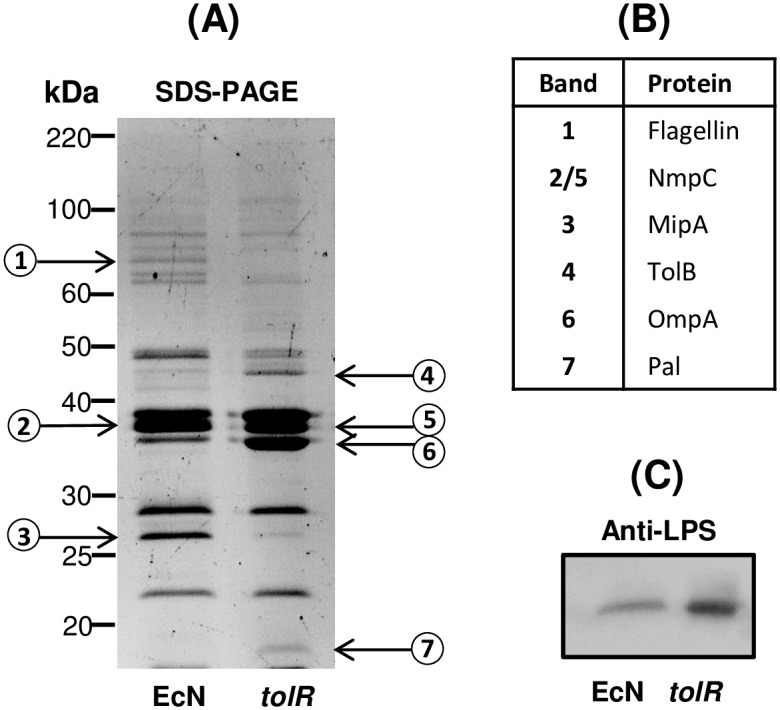
Protein profile and immunoblotting of LPS of MVs isolated from EcN and EcN *tolR* strains. **(A)** Comparison of the protein profile of MVs from EcN and EcN *tolR*. Isolated vesicles (10 μg protein) were separated in a 10%-SDS-PAGE gel and stained with Sypro^®^ Ruby Protein Gel Stain. Molecular size markers are indicated. Seven protein bands (labelled by numbers) were excised from the gel and analyzed by LC-MS/MS (data from these analyses are provided in S1 Table). **(B)** The name of the protein with the highest score is indicated for each band. **(C)** Western blot analysis of LPS in MVs isolated from EcN and EcN *tolR* strains. MV samples (0.1μg protein) were separated in a 15%-SDS-PAGE gel and analysed with specific anti-*E*. *coli* LPS antibodies. Representative SDS-PAGE and blots from three independent experiments are shown.

To further characterize the MVs isolated from the EcN *tolR* mutant, the protein profile was compared to the wild-type EcN strain by SDS-PAGE (Fig 2A). Most of the protein bands were invariably present in both samples, although some differences were observed. Seven differential protein bands were excised from the gel, subjected to in-gel trypsin digestion and analyzed by LC- MS/MS (S1 Table). The name of the protein displaying the highest score in each band is indicated in Fig 2B. The results showed that the most relevant differences were correlated with disturbances in outer membrane structures. MVs isolated from the *tolR* mutant were enriched in the peptidoglycan-associated lipoprotein Pal and TolB (bands 4 and 7), but deficient in flagellin and the murein-interacting protein MipA (bands 1 and 3). In both EcN and EcN *tolR* MVs the three main protein bands, close to the 40 kDa marker, corresponded to the outer membrane proteins OmpC, OmpF, NmpC and OmpA. However, the protein displaying the lowest molecular mass (mainly OmpA) was diminished in the wild-type MVs (band 6).

We also performed Western blot analyses of LPS in both MV samples (Fig 2C). At equal protein amounts, the LPS content of EcN *tolR* MVs was higher than that of the wild-type MVs. These results were consistent with the LPS concentration values calculated by the Purpald method (Table 1).

### High resolution TEM of EcN and EcN *tolR*

**Fig 3:**
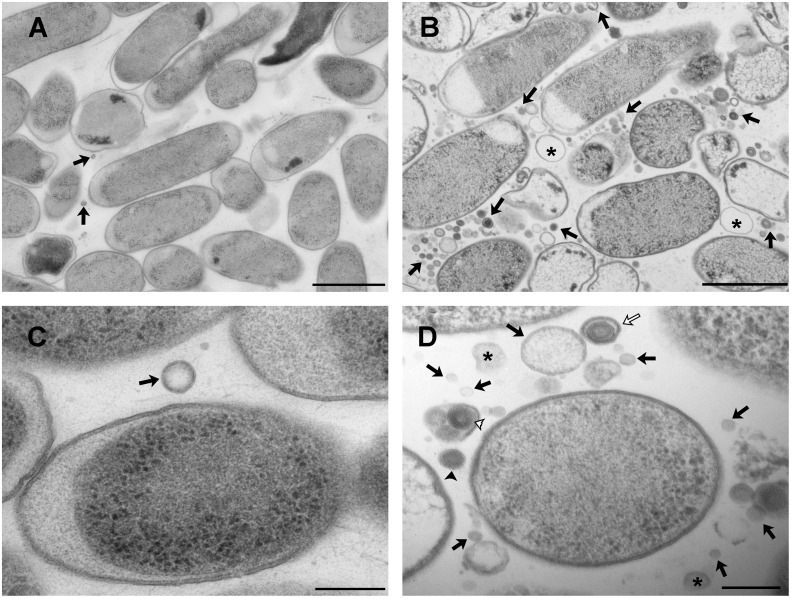
TEM micrographs of ultrathin sections from EcN and EcN *tolR* strains prepared by HPF-FS. **(A)** A representative micrograph of EcN cells in which few MVs are observed (arrows). **(B)** A representative micrograph of EcN *tolR* cells in which a huge amount of MVs can be observed interspersed among cells (arrows). Asterisks indicate bigger empty vesicles. **(C)** A magnified view of EcN cells in which a common OMVs with one bilayer is observed (arrow). **(D)** A magnified view of EcN *tolR* cells in which the following types of MVs are seen: common MVs (OMVs) indicated by black arrows; two bilayer vesicles (O-IMVs) by black arrow heads; multi-layered vesicles by white arrows; grouped vesicles by white arrow heads, and partially circularized membranes by asterisks. Representative images of thin-sections from two different experiments are shown. Bars A–B are 1μm, C–D are 200 nm.

To evaluate whether mutation in the Tol-Pal complex induced changes in the ultra-structure of EcN and its MVs, EcN and EcN *tolR* strains were examined by TEM following HPF-FS. Analysis of TSA solid cultures revealed important differences in the amount and morphology of MVs released into the extracellular space. For EcN, few MVs were observed between cells (Fig 3A, arrows), while in sections of EcN *tolR* a huge amount of spherical structures appeared, mainly interspersed among cells (Fig 3B, arrows), confirming its hypervesiculating phenotype. After examining 30 fields in sections from wild-type and mutant strains in two replicates, the number of MVs in each field was quantified using markers from the software ImageJ-win64, and the mutant strain was found to produce 94.7 ± 27.4-fold more MVs than the wild-type strain.

The morphology of MVs differed notably between the strains. Most of those produced by EcN corresponded to the commonly named outer membrane vesicles (OMVs). Deriving from the cell outer membrane, OMVs are surrounded by a bilayer membrane and entrap cytoplasmic content, and have diameters smaller than 200 nm (Fig 3C, arrow heads). However, the EcN *tolR* mutant produced different types of MVs: some corresponded to the OMV type, with a normal variation in diameter between 20 and 200 nm (Fig 3D, arrows), but several atypical MVs were also detected. One type contained two bilayers, similarly to the outer-inner membrane vesicles (O-IMVs) previously described by our group [26] (Fig 3D, black arrow head). Another type showed three concentric bilayers (Fig 3D, white arrow). An additional singularity was the presence of small groups of between two and four MVs surrounded by a bilayer (Fig 3D, white arrow head). The diameter of the EcN *tolR* MVs was more variable and on average slightly bigger than that of EcN MVs. In some of the fields, larger (about 400 nm) and apparently empty MVs also appeared, which may correspond to re-annealed membranes from lysed cells (Fig 3B, asterisks). Additionally, fragments of bilayer membranes, partially circularized but not closed, were observed (Fig 3D, asterisks).

TEM observation of sections obtained after HPF-FS also revealed differences in cell morphology between EcN and EcN *tolR*. For EcN *tolR*, a high number of altered cells with variable morphology were perceived, including length, diameter and cell shape. Such differences have also been reported for *tolR* mutants of other Gram-negative bacteria [20,39,40].

### Cryo-TEM of MVs from EcN and EcN *tolR*

**Fig 4:**
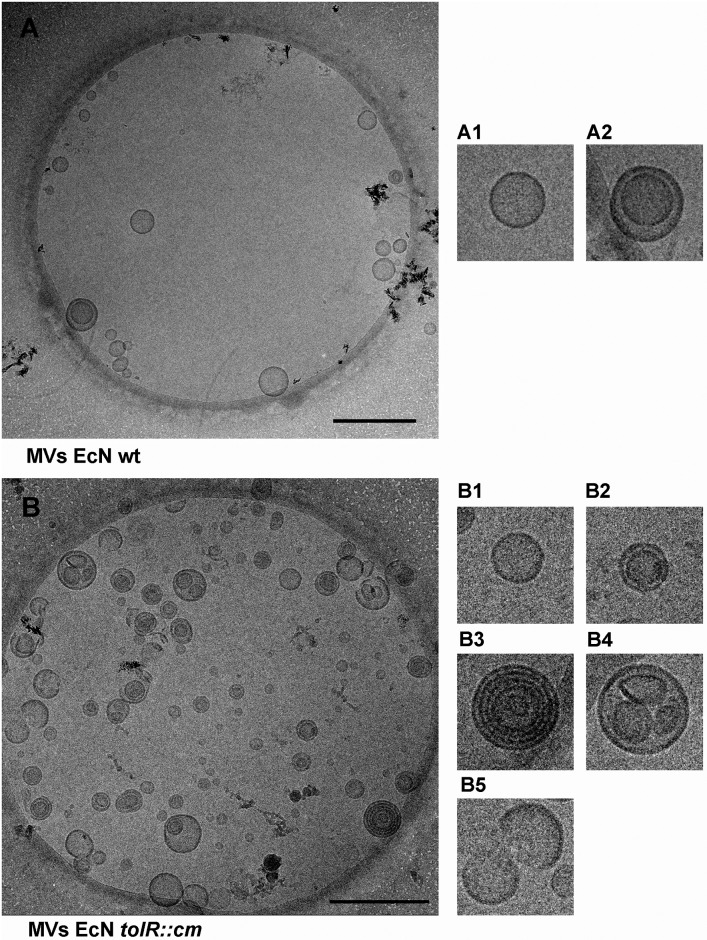
Isolated MVs from EcN and EcN *tolR* strains observed by cryo-TEM. **(A)** A representative image of EcN MVs, in which two types of MVs can be seen. The most abundant correspond to common OMVs (magnified view A1). A few of the recently described MVs named O-IMVs (magnified in view A2) were also observed. **(B)** A representive image of EcN *tolR* MVs showing different vesicle types. Magnified views are shown on the right: (B1) common OMVs; (B2) O-IMVs; (B3) multi-layered vesicles; (B4) grouped vesicles; (B5) partially circularized membranes. Representative images of plunge-frozen MVs from two different batches of MVs isolated from each strain are shown. Scale bars: 500 nm.

To further characterize MV structure, cryo-TEM analysis was performed. For this purpose, total MVs from both strains were isolated from liquid cultures, and the higher diversity in the types of MVs produced by EcN *tolR* was confirmed (Fig 4). In EcN samples (Fig 4A), single-bilayer OMVs predominated in all observed fields (Fig 4, A1), but double-bilayer vesicles (O-IMVs) (Fig 4, A2) were occasionally detected, as in the previous HPF-FS TEM observations. In contrast, EcN *tolR*-derived MVs analyzed by cryo-TEM showed a range of MV types, many of which did not correspond to the common OMVs or O-IMVs model (Fig 4B). In addition to OMVs (Fig 4, B1) and O-IMVs (Fig 4, B2), multilayered MVs were often observed (Fig 4, B3), as well as small groups of MVs surrounded by a bilayer (Fig 4, B4), and fragments of partially circularized bilayer membranes (Fig 4, B5). Altogether, the electron and cryo-electron microscopy studies confirmed the existence of substantial heterogeneity in the structure of MVs from the EcN *tolR* mutant.

### EcN *tolR*-derived MVs are not cytotoxic

MVs produced by EcN are not cytotoxic to HT-29 cells [37]. To test whether the different vesicular structures produced by the *tolR* mutant could affect cell viability, we examined the impact of EcN *tolR*-derived MVs on Caco-2 cell growth. To this end, kinetic studies were performed in Caco-2 cells exposed to EcN *tolR* MVs (5 μg/ml) or EcN MVs (as a control) for up to 168 h. Cell numbers were calculated by trypan blue exclusion assays carried out every second day during the experiment. Results showed that cell viability was not altered by treatment with EcN *tolR* MVs. No significant differences in the percentage of viable cells were observed after exposure to MVs compared to untreated controls during the incubation period, which was between 95% and 99% in all cases (S3 Fig).

### Uptake of EcN- and EcN *tolR*-derived MVs by intestinal epithelial cells

**Fig 5:**
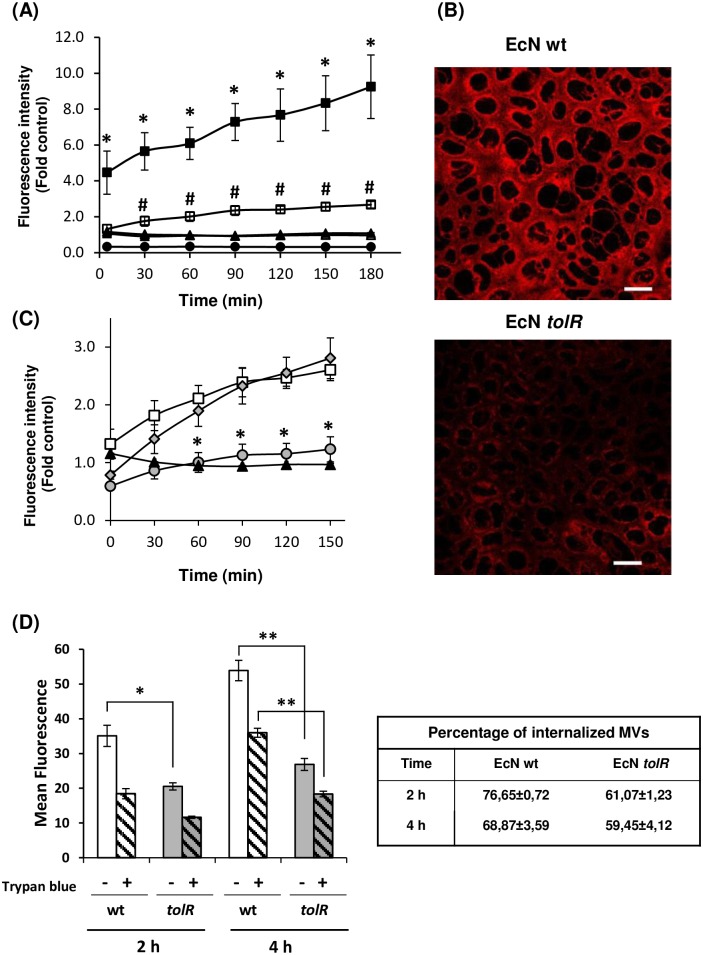
MV uptake by Caco-2 cells. **(A)** Rhodamine B-R18-labeled MVs (1 μg protein) from EcN (closed squares) or EcN *tolR* (open squares) were applied to polarized Caco-2 cells and fluorescence was measured over time. Caco-2 cells (circles) and MVs (triangles) alone were used as controls of background fluorescence. Values are means ± standard error from three independent experiments. Statistical differences were assessed using one-way ANOVA followed by Tukey’s test. *P<0.02, cells incubated with labeled MVs versus the background fluorescence emitted by MVs alone; # *P*<0.02, cells incubated with EcN *tolR*-derived MVs *versus* cells incubated with EcN MVs. **(B)** Visualization of internalized MVs by florescence microscopy. Caco-2 cells were incubated with rhodamine B-R18-labeled MVs for 1 and 3 h at 37°C. Internalized rhodamine B-R18-labeled MVs are visualized in red. Analysis was performed in a Leica TCS SP5 laser scanning confocal spectral microscope with a 63x oil immersion objective lens, and images were captured with a Nikon color camera (16 bit). Scale bars: 20 μm. **(C)** Internalization of EcN *tolR*-derived MVs in the presence of endocytosis inhibitors. Caco-2 cells were pre-incubated for 1h at 37°C with the lipid raft disrupting agent filipin III (gray diamonds) or with the clathrin-mediated endocytosis inhibitor chlorpromazine (gray circles) before adding rhodamine B-R18-labeled OMVs (1 μg/well) from the EcN *tolR* mutant. Uptake experiments were performed in the absence of endocytosis inhibitors for comparison (open squares). Fluorescence intensity was normalized by fluorescence detected at the indicated time points by labeled MVs in the absence of cells (black triangles). Data are presented as means ± standard error from three independent experiments. Statistical differences were assessed using one-way ANOVA followed by Tukey’s test. Values significantly different from those of cells incubated with MVs in the absence of endocytosis inhibitors are indicated by an asterisk (*p<0.02). **(D)** Caco-2 cells were incubated with DiO-labeled OMVs (100 μg/ml protein) from strains EcN and EcN *tolR* for the indicated times and fluorescence was measured using a flow cytometer before (total cell-associated OMVs) and after (internalized OMVs) trypan blue quenching. Data are expressed as means of fluorescence intensities from 10,000 cells after subtraction of background fluorescence of cells without OMVs (means ± standard error from three independent experiments). Statistical differences were assessed using one-way ANOVA followed by Tukey’s test. Significance between cells incubated with EcN *tolR*-derived MVs *versus* cells incubated with EcN MVs (*p≤0.02; ** p≤0.002). The table on the right shows the percentage of internalized MVs for each sample and incubation time (internalized / total cell-associated MVs).

We have recently shown that EcN MVs are internalized in Caco-2 cells [28,37]. The presence of several types of MV structures in the samples isolated from the mutant strain EcN *tolR* prompted us to analyze whether this heterogeneous population of MVs could be internalized by Caco-2 cells. To this end, MVs were labelled with rhodamine B-R18, whose fluorescence is quenched when intercalated into bilayer membranes at a high concentration. However, this dye fluoresces when diluted upon membrane fusion and internalization. As expected, no changes in fluorescence emission were observed in non-treated cells or samples containing only labelled MVs, whereas rhodamine B-R18-labelled EcN MVs (1 μg protein/well) applied to the apical side of differentiated Caco-2 cells produced a time-dependent increase in fluorescence. Interestingly, the fluorescence level observed in cells incubated with an equal amount of EcN *tolR* MVs was significantly lower (Fig 5A; # *p*<0.02). Confocal fluorescence microscopy analysis confirmed a reduced uptake by Caco-2 cells of the EcN *tolR*-derived MVs. Immunostaining of the peripheral membrane-associated protein ZO-1 was performed as an epithelial cell membrane marker (S4 Fig). Representative images captured under the same laser intensity for internalized EcN and EcN *tolR* vesicles after 1 h incubation with an equal amount of rhodamine B-R18-labelled MVs are shown in Fig 5B. Previous studies performed in several epithelial cell lines showed that MVs from wild-type EcN enter intestinal epithelial cells through clathrin-mediated endocytosis [37]. To check whether this endocytic pathway is also responsible for the uptake of EcN *tolR*-derived MVs, time-course internalization experiments using rhodamine B-R18-labeled MVs were performed in Caco-2 cells in the presence of inhibitors of endocytosis pathways. MV internalization was not reduced by disruption of lipid raft microdomains and caveolae by filipin III, but was drastically inhibited by chlorpromazine, an inhibitor of the clathrin-mediated pathway (Fig 5C). Thus, in spite of the heterogeneity in MVs produced by the *tolR* mutant, the entry pathway does not differ from that of wild-type MVs. As for EcN MVs, internalization of MVs from EcN *tolR* by undifferentiated Caco-2 cells was also specifically inhibited by chlorpromazine, but not by filipin III (not shown).

To confirm the different capacity of wild-type and *tolR*-derived MVs to become internalized by Caco-2 cells, we carried out flow cytometry experiments using MVs labelled with the fluorescent membrane dye DiO, which has different florescence properties from the lypophylic dye rhodamine B-R18. To distinguish internalized from cell surface-bound MVs, extracellular DiO-MV fluorescence was quenched with trypan blue. Results presented in Fig 5D show that DiO-labelled MVs from both strains were bound and internalized in Caco-2 cells in a time-dependent manner. However, the ability of MVs to bind and enter epithelial cells differed significantly between wild-type and *tolR* strains. The mean fluorescence intensities after 2 h and 4 h incubation were significantly lower for EcN *tolR* vesicles, thus confirming their reduced capacity for epithelial cell entry. Interestingly, the percentage of cell-associated MVs (in the absence of trypan blue) that are internalized (in the presence of trypan blue) did not significantly differ between wild-type and *tolR* MVs (Fig 5D). These results indicate that the lower internalization of EcN *tolR*-derived MVs could be explained by their reduced ability to bind epithelial cell membranes. Notably, even for the wild-type EcN, not all cell-associated MVs were internalized. It has been described that the abilities of MVs to bind and enter epithelial cells depend on the cell line used [41,42]. Studies performed with DiO-labelled MVs from enterohaemorrhagic *E*. *coli* show a lower proportion of internalized MVs in Caco-2 cells than in other cell lines [42], with fluorescence intensity values comparable to those measured here in Caco-2 cells incubated with EcN MVs.

## Discussion

The study of extracellular vesicles is an ongoing research area, not only in mammalian cells but also in bacteria [43]. MVs are considered intercellular communicasomes, as they act as a mechanism for distance delivery of active compounds between cells. In this context, MVs released by commensal bacteria are foreseen as key players in signaling processes in the intestinal mucosa [4]. Although still few, the reports on microbiota-produced MVs prove that they promote immunomodulatory effects in intestinal epithelial and immune cells, as well as *in vivo* models [28,44]. The ability of bacterial MVs to interact with and enter host cells has prompted the exploration of their potential for novel clinical and biotechnological applications [6,11,45].

As stated above, a major drawback of such applications of bacterial MVs is the very low yield of MVs isolated from *in vitro* cultures. One strategy to overcome this limitation is the use of cell envelope protein mutants with a hypervesiculating phenotype. In this context, mutants in the *tol-pal* system have been constructed in various bacterial species to increase MV production. Although analysis by negative stain-TEM has revealed that MVs released by *tol* mutants display the normal features of bacterial MVs, some differences in the protein content, immunogenicity, and pro-inflammatory properties have been reported for *H*. *pylori* Δ*tolB* and Δ*pal* mutants [20]. These findings clearly point to differences in the molecular composition of MVs isolated from *tol* mutants and call for a more in-depth knowledge of MV structure before their application in functional studies or for biotechnological purposes.

To gain further insight into the molecular structure of MVs released by *tol* system mutants, we used the probiotic strain EcN as a model. MVs from EcN and the derived *tolR* mutant were isolated after 15 h of growth, as functional studies are often performed with MVs collected from overnight cultures (from 12 to 15 h growth) [9,29,42,46–47]. As in *tolR* mutants derived from other Gram-negative bacteria [48], the EcN *tolR* growth curve was similar to that of the wild-type EcN. Although minor differences in the growth rate were observed during the early exponential phase, both strains exhibited equal cell numbers and OD_580_ values during the late exponential and early stationary phases. Highly vesiculating phenotypes have been described for many *E*. *coli tol-pal* mutants [13,19], so it was not surprising that EcN *tolR* exhibited significant increases in MV production compared to the wild-type strain, ranging from 32.9- to 77.8-fold, according to the quantification method. Relative values of vesicle production based on fluorescent lipid measurement were higher than with protein analysis, as reported in other studies [13]. Although the greater amounts of protein, LPS or lipids in the *tolR* mutant could be due to a larger vesicle size, the slight increase in *tolR* MV size observed was not enough to justify the 32.9-fold or higher increases measured with the three methods.

While the protein profile of EcN *tolR*-derived MVs was quite similar to that of the MVs isolated from the wild-type strain, some differences were identified. The most relevant differences correlate with disturbances in the outer membrane structures, likely resulting from TolR deficiency. Thus, MVs released by the *tolR* mutant displayed a higher content of TolB (periplasmic protein) and Pal (outer membrane protein), two proteins of the Tol-Pal complex. As formation of the heteromultimeric protein complex TolQRA-B-Pal is altered as a consequence of TolR deficiency, non-interacting TolB and Pal proteins could then be released through MVs. As for *Gallibacterium anatis tolR* mutants [48], the MVs produced by the EcN *tolR* mutant were enriched in OmpA. This abundant outer membrane protein also contributes (like the Tol-Pal system) to linking PG to the outer membrane. It has been suggested that increased OmpA levels may compensate for the membrane instability in Tol-Pal-deficient strains [48]. On the contrary, both the scaffold protein MipA required for PG biosynthesis and flagellin were underrepresented in EcN *tolR* MVs. A low production of flagella has also been reported for a *H*. *pylori tolB* mutant, and the released MVs similarly showed specific differences in protein composition with respect to those produced by wild-type *H*. *pylori*, with about ten proteins absent in those of the mutant strain [20]. Overall, we can conclude that the main alterations in the molecular composition of EcN *tolR*-derived MVs may be attributed to TolR deficiency.

The composition of MVs obtained from *tolR* mutants has been studied by several groups, but the MV structure has generally not been analyzed in detail. TEM observation of negatively stained MVs is the most commonly used technique to assess MV presence and morphology, but it does not provide enough resolution to distinguish between different types of MVs, artifacts, or re-circularized membranes from lysed cells [13,19,22,23,49,50]. Some studies have described vesicle morphology by scanning electron microscopy (SEM) or thin-section TEM of chemically fixed and dehydrated samples at room temperature [14,15,51–53]. However, in both methods the extracellular matter, which includes MVs, has a marked propensity to collapse and be removed during sample preparation [2,54]. Surprisingly, few studies have used TEM of cryo-immobilized specimens by HPF followed by FS, or cryo-TEM to visualize MV samples, although both have proven very useful for observing detailed structures of MVs and their producing strains [26,27,34,55,56].

In this study, when TEM of negatively stained MVs was used to compare structural differences between vesicles from EcN and EcN *tolR*, only a slight increase in the mean diameter of EcN *tolR* vesicles was observed and a lower small vesicle population; no details other than their spherical shape and size were appreciated with this technique. Conversely, TEM observation of thin-sections of HPF-FS samples from both strains allowed us to visualize the higher MV production by EcN *tolR*, and that different types of MVs were interspersed among cells. The presence of different types of MVs was further confirmed by cryo-TEM observation of plunge frozen-hydrated isolated MVs, which is the less artifactual technique. In EcN samples, two types of MVs were observed, most corresponding to common OMVs and a few to the recently described O-IMVs [26,27]. On the other hand, cryo-TEM of EcN *tolR* samples revealed at least five types of hypothetical MVs. The presence of different types of MVs, including trilayered MVs, or whorled outer membrane fragments, has also been confirmed in an ultrastructural analysis of MVs from *Helicobacter pylori* strain 60190 [52].

The protein / fluorescent lipid ratio did not return the same value for EcN and EcN *tolR* MVs, the *tolR* mutant ratio being less than half that of the wild-type strain. This lack of correlation can be explained by the different types of MVs produced by the *tolR* mutant, in which the protein-to-lipid ratio was not maintained when compared with wild-type MVs. TEM and cryo-TEM observations confirmed both the over-vesiculation phenotype of EcN *tolR* and the variety of shapes of *tolR* MVs. Considering the morphological diversity of MVs, many of which had multilayered lipid bilayers, it seems reasonable that the protein-to-lipid ratio is lower in *tolR* MVs. Analyzing MVs structure by TEM after HPF-FS and cryo-TEM techniques, instead of relying only on electron microscopy of negative stained MVs, allowed us to obtain complementary and more accurate information about the over-vesiculating EcN mutant strain.

Although it cannot be discarded that some cell lysis may have contributed to the high amount of MVs produced by the *tolR* mutant, we can rule out that the over-vesiculation and changes in MV morphology were due to impaired growth or cell lysis, since both strains exhibited normal growth with identical cell counts and OD_580_ at the time of vesicle collection. One hypothesis is that MVs from EcN *tolR* are unstable and break easily. Rennelli and co-workers [56] explored this possibility by cryo-TEM and established that only 7.3% of MVs from *Pseudomonas aeruginosa* PAO1 were broken. A similar proportion was observed in EcN mutant MVs, which would not explain the marked increase in vesiculation.

Our study shows that not only can the composition and structure of MVs be modified by mutations in the cell envelope proteins, but their interaction with target cells can also be affected. Analysis of MV uptake, measured by the increase in rhodamine-emitted fluorescence and microscope imaging, indicated a reduced level of internalized vesicles derived from the EcN *tolR* mutant. Quantification of the fluorescence emitted upon internalization in Caco-2 cells allowed us to estimate a roughly 3-fold reduction in the number of intracellular MVs in comparison with wild-type-derived MVs. Interestingly, *tolR*-derived MVs are internalized by the same endocytic pathway as EcN MVs, as their uptake is impaired in the presence of the clathrin-mediated endocytosis inhibitor chlorpromazine. Flow cytometry analysis performed with DiO-labelled OMVs confirmed the lower internalization values for *tolR*-derived MVs. In addition, fluorescence intensity values before and after trypan blue addition allowed us to establish that the low number of internalized MVs was due to a lower cell binding capacity of the *tolR*-derived MVs, rather than a different entry pathway or mechanism. These results suggest that only certain types of MVs, most likely conventional OMVs and O-IMVs, efficiently interact with their target(s) or receptor(s) in the cell membrane, a key step before being taken up by epithelial cells through clathrin-mediated endocytosis.

## Conclusions

High yields in MV production are desirable if the MVs are to be used for functional studies or biotechnological purposes. Independently of the strategy used to increase production yield, there is a need to obtain a well-defined and uniform pool of MVs. Our study confirms that the introduction of a *tolR* mutation in the probiotic EcN induces a hypervesiculation phenotype. MVs retrieved from the mutant strain showed alterations in composition and in their ability to interact with host cells, which can be explained by significant modifications in MV structure. Production of different types of MVs or outer membrane structures by *tolR* mutants cannot be detected by TEM of negatively stained MVs, although this heterogeneity may have a major impact on MV functionality. This study evidences the need for conducting a detailed structural analysis by high resolution TEM techniques when working with hypervesiculating mutants. This analysis is crucial to improve and standardize the MVs used for therapy purposes.

## Supporting Information

S1 Fig PCR confirmation of *tolR* disruption in EcN. The mutant strain EcN *tolR* was constructed by P1-transduction from *E*. *coli* strain TPS300 (*tolR*::*Ωcm*). The correct integration of the *tolR*::*Ωcm* marker in the EcN genome was assessed by PCR amplification with the primers flanking *tolR* sequences: *FW-tolR* (TGCGCCGGAAGCCGTAGTGG) and *RV-tolR* (CCGCTTGTTTCTCACGCAGT). The size of the amplified products is indicated on the left. The increase in the size of the PCR product in the EcN *tolR* mutant confirms *tolR* disruption by the chloramphenicol cassette as in the donor strain TPS300.
(PDF)

S2 Fig Growth curves of EcN (cross) and EcN *tolR* (triangles) cultivated in LB medium monitored by viable counts (colony forming units, CFU/ml). (TIF)

S3 Fig Effect of EcN and EcN *tolR*-derived OMVs on viability of Caco-2 cells. Cell viability of Caco-2 cells exposed to OMVs (5 μg/ml) from EcN (blue) or EcN *tolR* (orange) for up to 7 days, measured by the trypan blue exclusion assay. Untreated Caco-2 cells (gray) were analyzed in parallel as a control. Values are means ± standard error from three independent experiments. Lack of statistical differences was confirmed by one-way ANOVA followed by Tukey’s test.
(TIF)

S4 Fig Visualization of internalized MVs by fluorescence microscopy. Caco-2 cells were incubated with rhodamine B-R18-labeled MVs, isolated from the indicated strains, for 1 hour at 37°C. The cell membrane was visualized by immunostaining with antibodies against the zonula occludens ZO-1 protein followed by Alexa Fluor 488-conjugated secondary antibody (green). Nuclei were stained with DAPI (blue). Internalized labeled MVs are visualized in red. Representative images from three independent experiments are shown. Scale bars: 20 μm.
(TIF)

S1 Table Proteomic analysis by LC-MS/MS. Identification of gel-excised seven protein bands labelled by numbers in Fig 2, which were differentially expressed between EcN and EcN *tolR*.
(XLSX)
