# Supplementary figures and images for: Membrane Vesicles Released by a hypervesiculating Escherichia coli Nissle 1917 tolR Mutant Are Highly Heterogeneous and Show Reduced Capacity for Epithelial Cell Interaction and Entry
Source: PLoS One. 2016 Dec 30;11(12):e0169186. doi: 10.1371/journal.pone.0169186 (PMC5201253; doi:10.1371/journal.pone.0169186)

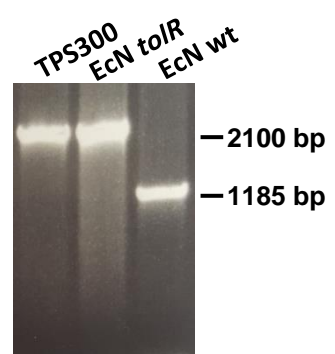

Supplement: S1 Fig — The mutant strain EcN tolR was constructed by P1-transduction from E. coli strain TPS300 (tolR::Ωcm). The correct integration of the tolR::Ωcm marker in the EcN genome was assessed by PCR amplification with the primers flanking tolR sequences: FW-tolR (TGCGCCGGAAGCCGTAGTGG) and RV-tolR (CCGCTTGTTTCTCACGCAGT). The size of the amplified products is indicated on the left. The increase in the size of the PCR product in the EcN tolR mutant confirms tolR disruption by the chloramphenicol cassette as in the donor strain TPS300. (PDF) [file pone.0169186.s001.pdf]

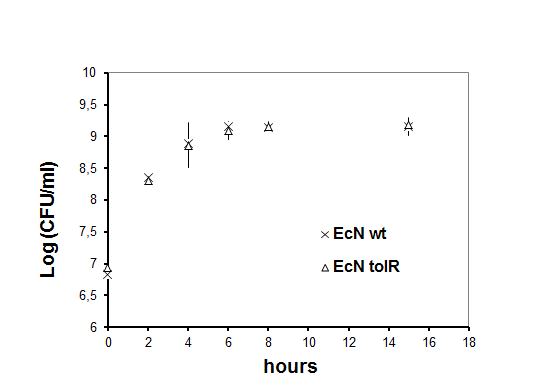

Supplement: S2 Fig — (TIF) [file pone.0169186.s002.tif]

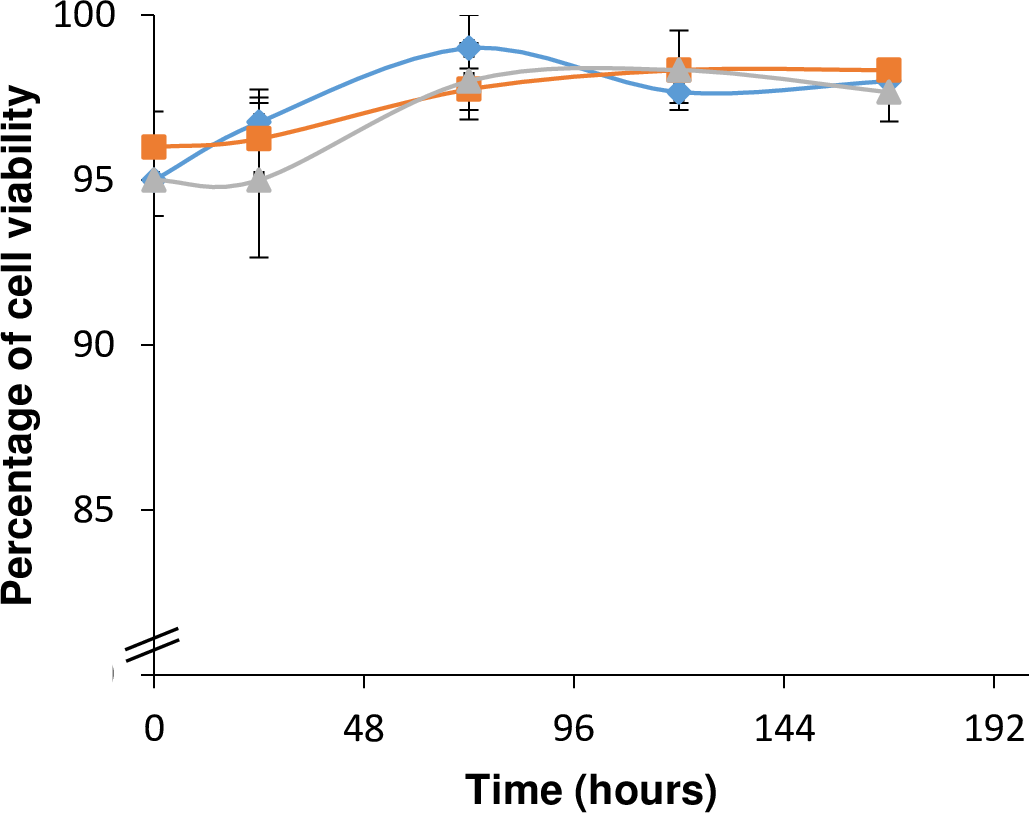

Supplement: S3 Fig — Cell viability of Caco-2 cells exposed to OMVs (5 μg/ml) from EcN (blue) or EcN tolR (orange) for up to 7 days, measured by the trypan blue exclusion assay. Untreated Caco-2 cells (gray) were analyzed in parallel as a control. Values are means ± standard error from three independent experiments. Lack of statistical differences was confirmed by one-way ANOVA followed by Tukey’s test. (TIF) [file pone.0169186.s003.tif]
